# Dimensional Model in Personality Disorders: A Comparative Analysis

**DOI:** 10.1192/j.eurpsy.2026.11465

**Published:** 2026-07-31

**Authors:** P. Juncosa Montes, G. Villarreal Orellano, A. Vazquez Vazquez

**Affiliations:** 1 Hospital Universitario de Jerez de la Frontera, Unidad de Salud Mental de Sanlucar; 2 Hospital Universitario de Jerez de la Frontera, Unidad de Salud Mental de Jerez, Jerez de la Frontera, Spain

## Abstract

**Introduction:**

Borderline Personality Disorder (BPD) has been associated with early adverse experiences and difficulties in self-regulation (*Zashchirinskaia et al. Iranian Journal of Psychiatry; 18(1), 65-71).*

**Objectives:**

This study compares childhood trauma, self-care, and clinical symptoms across three groups: individuals diagnosed with BPD, with Adjustment Disorder (AD), and individuals without psychiatric diagnoses. A dimensional approach is proposed to explore whether AD represents a milder form of dysfunction. Group differences, correlations between trauma and self-care, and the predictive value of trauma on symptom severity in BPD were analyzed.

**Methods:**

A total of 90 participants were included (30 per group). The following instruments were administered: the EARLY scale (childhood trauma), SCPRS-R (self-care), and BSL-23 (BPD symptomatology). MANOVA analyses, Spearman correlations, and linear regression were conducted. Data collection took place in a community mental health unit, following ethical standards and obtaining informed consent.

**Results:**

The BPD group reported higher levels of trauma (emotional neglect, abuse, excessive demands) and lower self-care (physical care, healthy behaviors, boundary setting), followed by the AD group. The non-clinical population showed the most favorable levels. Negative correlations were found between trauma and self-care. In the BPD group, childhood trauma explained 50% of the variance in clinical severity (adjusted R² = .506), although no specific trauma subtype emerged as a unique significant predictor.

**Conclusions:**

Findings support a dimensional model of personality functioning: BPD as a severe form, AD as moderate dysfunction, and the non-clinical population as a healthy reference. Childhood trauma, particularly emotional in nature, is associated with poorer self-care and increased symptom severity (*Hepp et al. 2021; 12(1), 29–38).* Assessing these variables may enhance early detection and the personalization of clinical interventions in mental health care.

**Disclosure of Interest:**

None Declared

